# Biological characterization of chemically diverse compounds targeting the *Plasmodium falciparum* coenzyme A synthesis pathway

**DOI:** 10.1186/s13071-016-1860-3

**Published:** 2016-11-17

**Authors:** Sabine Fletcher, Leonardo Lucantoni, Melissa L. Sykes, Amy J. Jones, John P. Holleran, Kevin J. Saliba, Vicky M. Avery

**Affiliations:** 1 Discovery Biology, Eskitis Institute for Drug Discovery, Griffith University, Nathan, QLD Australia; 2 Medical School and Research School of Biology, The Australian National University, Canberra, ACT Australia

**Keywords:** Coenzyme A synthesis, *Plasmodium falciparum*, Gametocytes, *Trypanosoma﻿ brucei brucei*, *Trypanosoma cruzi*, Drug discovery

## Abstract

**Background:**

In the fight against malaria, the discovery of chemical compounds with a novel mode of action and/or chemistry distinct from currently used drugs is vital to counteract the parasite’s known ability to develop drug resistance. Another desirable aspect is efficacy against gametocytes, the sexual developmental stage of the parasite which enables the transmission through *Anopheles* vectors. Using a chemical rescue approach, we previously identified compounds targeting *Plasmodium falciparum* coenzyme A (CoA) synthesis or utilization, a promising target that has not yet been exploited in anti-malarial drug development.

**Results:**

We report on the outcomes of a series of biological tests that help to define the species- and stage-specificity, as well as the potential targets of these chemically diverse compounds. Compound activity against *P. falciparum* gametocytes was determined to assess stage-specificity and transmission-reducing potential. Against early stage gametocytes IC_50_ values ranging between 60 nM and 7.5 μM were obtained. With the exception of two compounds with sub-micromolar potencies across all intra-erythrocytic stages, activity against late stage gametocytes was lower*.* None of the compounds were specific pantothenate kinase inhibitors. Chemical rescue profiling with CoA pathway intermediates demonstrated that most compounds acted on either of the two final *P. falciparum* CoA synthesis enzymes, phosphopantetheine adenylyltransferase (PPAT) or dephospho CoA kinase (DPCK). The most active compound targeted either phosphopantothenoylcysteine synthetase (PPCS) or phosphopantothenoylcysteine decarboxylase (PPCDC). Species-specificity was evaluated against *Trypanosoma cruzi* and *Trypanosoma brucei brucei*. No specific activity against *T. cruzi* amastigotes was observed; however three compounds inhibited the viability of trypomastigotes with sub-micromolar potencies and were confirmed to act on *T. b. brucei* CoA synthesis*.*

**Conclusions:**

Utilizing the compounds we previously identified as effective against asexual *P. falciparum*, we demonstrate for the first time that gametocytes, like the asexual stages, depend on CoA, with two compounds exhibiting sub-micromolar potencies across asexual forms and all gametocytes stages tested. Furthermore, three compounds inhibited the viability of *T. cruzi* and *T. b. brucei* trypomastigotes with sub-micromolar potencies and were confirmed to act on *T. b. brucei* CoA synthesis, indicating that the CoA synthesis pathway might represent a valuable new drug target in these parasite species.

**Electronic supplementary material:**

The online version of this article (doi:10.1186/s13071-016-1860-3) contains supplementary material, which is available to authorized users.

## Background

Malaria is a tropical infectious disease caused by intracellular apicomplexan parasites of the genus *Plasmodium*, which are transmitted to the human host during the blood meal of an infected female *Anopheles* mosquito. Half of the world’s population is at risk of contracting the disease and over 200 million cases are reported annually, of which more than 400,000 are fatal [1]. Malaria is curable and a dramatic reduction in mortality rates has been achieved in the last decade, thanks to sustained efforts by multiple donor agencies and the WHO [2]. However, the development of parasite resistance to chemotherapeutics remains a major concern. Resistance against classic antimalarials, such as chloroquine and pyrimethamine, is widespread and has severely reduced the efficacy of these drugs [3]. Alarmingly, development of resistance against the current drug of choice, artemisinin, which is the core compound of the widely used artemisinin combination therapies (ACT), has recently been reported in four Southeast Asian countries and appears to be spreading [4, 5]. To prevent a lack of effective therapeutics in the future, new anti-malarial compounds, ideally acting on different targets and/or displaying novel mechanisms of action, urgently need to be identified and developed [3, 6].

**Fig. 1:**
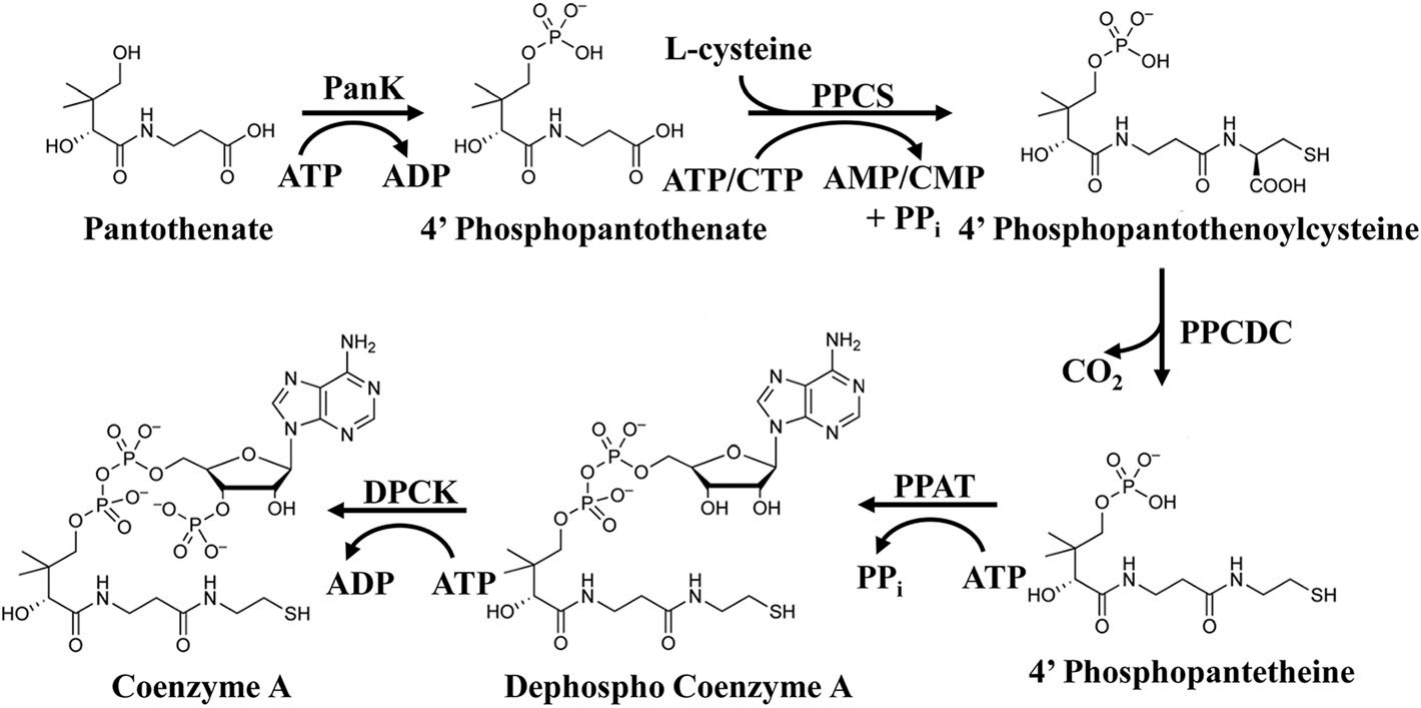
Enzymatic steps of the *P. falciparum* CoA synthesis pathway. *Abbreviations*: PanK, pantothenate kinase; PPCS, phosphopantothenoylcysteine syntetase; PPCDC, phosphopantothenoylcysteine decarboxylase; PPAT, pantetheinephosphate adenylyltransferase; DPCK, dephospho coenzyme A kinase

Coenzyme A (CoA) plays a central role in eukaryotic metabolism as an acyl carrier. Its acetylated form, acetyl-CoA, enters the tricarboxylic acid (TCA) cycle, a central metabolic hub. It serves as a vital co-factor for fatty acid synthesis [7], as well as pyruvate and fatty acid oxidation [8] for energy production in the form of ATP [9]. CoA is synthesized in five enzymatic steps (Fig. 1) from units derived from pantothenic acid (vitamin B_5_), ATP and cysteine. Pantothenate kinase (PanK) catalyses the first step of the *Plasmodium falciparum* CoA synthesis pathway, phosphorylation of pantothenate to 4′-phosphopantothenate. In the second synthesis step, an L-cysteine molecule is integrated by phosphopantothenoylcysteine synthetase (PPCS) and the resulting intermediate, 4′- phosphopantothenoylcysteine, is decarboxylated to 4′-phosphopantetheine by the third enzyme, phosphopantothenoylcysteine decarboxylase (PPCDC). Phosphopantetheine adenylyltransferase (PPAT) catalyses the penultimate step of the synthesis, converting 4′-phosphopantetheine into dephospho-CoA (dP-CoA). The final phosphorylation step that completes CoA synthesis is catalyzed by dP-CoA kinase (DPCK). In humans, this enzyme is not a single entity but is linked to phosphopantetheine adenylyltransferase (PPAT) to form a bifunctional enzyme that can be regarded as a CoA synthetase [10]. Despite conservation of function, the coding sequences of the enzymes involved in CoA synthesis are not highly conserved between eukaryotic species [11] and the essentiality of several of the *P. falciparum* enzymes has been predicted in two independent *in silico* studies based on metabolic network analysis [12, 13]. This fact presents an opportunity to target specifically the *P. falciparum* CoA synthesis pathway for the development of novel antimalarial drugs.

In 2005, pantothenol (provitamin B_5_) was identified as an inhibitor of *P. falciparum* growth [14]. It was shown to interact with pantothenate kinase (PanK) and its mechanism of action attributed to an effect on CoA synthesis or utilization [14]. Subsequent work also demonstrated that intra-erythrocytic *P. falciparum* is capable of *de novo* CoA synthesis, consistent with parasite survival being independent of host CoA biosynthesis [15]. The investigation of a series of pantothenate analogues revealed several compounds with modest anti-plasmodial activity [16]. Recently, pantothenamides (secondary or tertiary amides of pantothenic acid) were shown to inhibit *P. falciparum* proliferation with sub-micromolar activity, but only when the serum enzyme pantetheinase is inhibited [17]. Currently, different strategies are being developed to overcome pantetheinase-mediated degradation of pantothenamides, thereby improving the activity of this group of pantothenic acid analogs in vivo [18–21].

Utilizing an alternative approach, rather than chemically modifying a specific substrate of the CoA synthesis pathway, we recently developed a CoA chemical rescue screening approach to identify novel, chemically diverse inhibitors of the CoA pathway in asexual blood stage *P. falciparum* [22]. Supplementing the parasite culture medium with CoA allowed asexual *P. falciparum* forms to survive the anti-plasmodial effect of eleven chemically-diverse inhibitors, consistent with these compounds inhibiting CoA synthesis or utilization. The inhibitors were identified from two small chemical compound libraries, one in-house library and the Medicines for Malaria Venture (MMV) Malaria Box [23]. In this study we report the further characterization of the stage- and species-specificity of the compounds that showed the most prominent rescue by CoA in our previous report. Firstly, stage specificity was assessed on gametocytes, the sexual stages of *P. falciparum*, which emerge among the intra-erythrocytic population and develop over 10–12 days into 5 morphologically different stages (I to V) [24]. Gametocytes are essential for inter-host transmission of malaria [25]. Previous research demonstrated that gametocyte stages show remarkable differences to the asexual blood stages of the parasites at the cellular, transcriptional and metabolic levels [25–28], and in their sensitivity to antimalarial drugs [29, 30]. Secondly, the species-specificity was assessed using two other protozoan parasites, namely *Trypanosoma cruzi* and *Trypanosoma brucei brucei*. Potential targets of the compounds were examined through assessment of parasite growth rescue patterns after supplementation with different CoA pathway intermediates.

## Results

### Compound activity on *Plasmodium falciparum* sexual development stages

#### Compound activity against early and late stage gametocytes

All test compounds displayed gametocytocidal activity. Similar IC_50_ activities were observed on early stage gametocytes to those previously reported for the asexual blood stage forms of the parasite (Additional file 1: Table S1).

The IC_50_ values obtained for the compounds against early stage gametocytes ranged between 60 nM and 7.5 μM, with four compounds displaying sub-micromolar activity (Additional file 1: Table S1). Against the later stages (IV and V) of gametocyte development, three compounds showed significantly reduced activity compared to both asexual stages and early stage gametocytes (Additional file 1: Table S1). Compounds Amb4317088, STK 668036 and MMV665980 displayed moderate, single digit micromolar activity against all development stages tested. Very promising results were obtained for STK 740987 and Amb180780, which had sub-micromolar IC_50_ values on all three *P. falciparum* developmental stages investigated (Additional file 1: Table S1). The IC_50_ range for STK 740987 was 0.38–0.46 μM across all *P. falciparum* life-cycle stages tested, with an overall SI over human embryonic kidney cells (HEK293) above 94. The IC_50_ range for Amb180780 was 0.06–0.12 μM across the tested life-cycle stages, with overall HEK293 SI > 482.

#### Gametocyte-infected red blood cell deformability

The lipid composition of the RBC membrane can influence the deformability of the cell, as observed under treatment with 3-hydroxy-3-methylglutaryl CoA reductase (HMG-CoA reductase) inhibitors such as simvastatin, pravastatin and lovastatin [31–33]. Since CoA plays a vital role in the synthesis and oxidation of fatty acids in general [9] and is furthermore directly required for the synthesis of 3-hydroxy-3-methylglutaryl-CoA, the main substrate of HMG-CoA reductase, we were interested in investigating whether any of the CoA synthesis inhibitors demonstrating late stage gametocytocidal activity would exhibit an effect on RBC deformability. The ability of late stage gametocyte-infected RBC to cross a spleen mimic was assessed using an optimised microsphere filtration assay based on a recently published approach [34]. The retention of gametocyte-infected RBCs was determined after treatment for 2 or 24 h with compound concentrations ranging from 0.1 nM to 10 μM. Since dead parasites also display a higher retention rate in the filtration assay, the viability of the compound-treated gametocytes was assessed simultaneously using MitoTracker ® Red staining and confocal microscopy. No increased retention of late stage gametocytes in the spleen mimic were observed that did not coincide with parasite death for any of the test compounds. Therefore, no specific deformability changes, independent of gametocytocidal activity, could be determined for the CoA synthesis inhibitors examined (Additional file 1: Figure S1).

### Compound activity against *Trypanosoma cruzi* and *Trypanosoma brucei brucei*

The next step taken was to determine whether the activity of the compounds was confined to *P. falciparum,* or whether it extended to other protozoan parasites. Compound activity against two members of another clinically important group of parasites, the kinetoplastids *T. cruzi* and *T. b. brucei* was determined. The sensitive high content imaging assay used to determine compound activity against *T. cruzi* assesses the intracellular amastigote life form of the parasite in the 3T3 host cell [35]. It is important to simultaneously assess compound activity against 3T3 cells to determine potential cytotoxicity against the host cell line, since this could result in a false positive outcome. The *T. b. brucei* assay employs the REDOX indicator Alamar Blue to assess viability, which was developed into a 384-well assay format [36].

The activity of the MMV malaria box compounds (MMV665820 and MMV665980) against *T. b. brucei* and *T. cruzi* has previously been reported in ChEMBL [37]. In our models, none of the compounds tested showed appreciable selectivity against *T. cruzi* amastigotes, compared to the 3T3 host cell line (Additional file 1: Table S1).

*Trypanosoma brucei brucei* causes African trypanosomiasis in animals and is not infective to humans. However, it is closely related to the human infective species *T. brucei gambiense* and *T. brucei rhodesiense* and is widely used as a surrogate organism to investigate anti-trypanosomal compound activity. As *T. b. brucei* trypomastigotes are extracellular blood stream parasites, no direct host cell comparison is required. As opposed to the negative outcome obtained with *T. cruzi*, several of the compounds investigated showed activity against *T. b. brucei*. Three compounds, namely STK 740987, Amb3377585 and Amb4317088, had very promising activities with IC_50_ values as low as 1 μM, 200 nM and 160 nM, respectively (Additional file 1: Table S1), and one compound (MMV665820) was moderately active with an IC_50_ value of 2.5 μM. STK 740987 demonstrates a good selectivity index of > 39 for *T. b. brucei* compared to HEK293 cells. The selectivity indices of Amb4317088 and Amb3377585 are also promising, with values of 15 and 14, respectively (Additional file 1: Table S1).

The higher activity of the compounds against *T. b. brucei*, as opposed to the generally low activity observed on *T. cruzi* amastigotes, prompted us to investigate whether this discrepancy was due to species specific differences or due to the different trypanosome life forms used in the respective assays. Testing of the compounds in our recently developed *T. cruzi* trypomastigote assay [35], revealed higher compound activities against the extracellular trypomastigote form (Additional file 1: Table S1). The three most active compounds Amb4317088, STK 740987 and Amb3377585 displayed sub-micromolar IC_50_ values against *T. cruzi* trypomastigotes [0.392 μM, 0.682 μM and 0.744 μM, respectively (Additional file 1: Table S1)].

### Target investigation within the coenzyme A synthesis pathway

After the assessment of the broader biological activity profiles of the candidate compounds, we endeavored to identify if specific steps within the CoA synthesis pathway were affected by the inhibitors. To this end, we assessed a possible direct inhibition of the first enzyme of the pathway, PanK. Subsequently, given the current lack of direct enzymatic assays to measure the inhibition of the following biosynthetic steps, several metabolites of the pathway were tested on each parasite species and/or stage that had shown susceptibility to the experimental compounds, to ascertain if they could rescue the growth inhibitory effect caused by the compounds. Parasites were treated with the test compounds at their respective IC_80_ concentrations in the presence or absence of either pantothenate, pantethine, dP-CoA or CoA.

Pantothenate is the first substrate of the pathway (Fig. 1) and its addition was intended to rescue the action of possible competitive inhibitors of the first enzyme, PanK. Such a competitive inhibitory effect has previously been demonstrated for a number of pantothenate analogues, including pantothenol [16], herein used as a positive control compound for this assay. Pantethine is the dimer of pantetheine and not a direct intermediate of the pathway. It is known that bacteria are able to convert pantethine to pantetheine and to subsequently phosphorylate it to 4′phosphopantetheine, the substrate of PPAT, the fourth enzyme in the pathway (Fig. 1) [38]. It is not known, whether *P. falciparum* is capable of pantetheine phosphorylation for utilization in CoA synthesis. It was anticipated that if pantethine could be taken up by the parasite, reduced to pantetheine and then phosphorylated, pantethine would allow the parasites to bypass inhibition caused by inhibitors of the first three enzymes, namely PanK, PPCS and PPCDC. This possibility was investigated by testing whether pantethine was able to rescue the growth inhibitory effect of any of the inhibitors. Finally, the rescue ability of dP-CoA, the final substrate of CoA synthesis, was tested. Supplementation of dP-CoA was assumed to be able to rescue inhibition of any of the enzymatic steps, except for the final one (Fig. 1), allowing the identification of non-competitive DPCK inhibitors and downstream CoA utilizing enzymes, as such compounds are the only ones whose inhibition would not be rescued by dP-CoA. Inhibitors competing with dP-CoA for DPCK binding were expected to be displaced, thereby rescuing parasite survival, by dP-CoA supplementation in a concentration dependent manner.

#### Pantothenate kinase inhibition

A radiometric assay, which allows measurement of the phosphorylation of various substrates by kinases present in *P. falciparum* lysates [16, 39], was used to test the effect of the chemical compounds of interest at 100 μM concentration on PanK activity. Only one compound, STK 668036, showed a statistically significant PanK inhibition (93 ± 9%; Additional file 1: Figure S2a). This compound was also tested at three concentrations (2, 10 and 100 μM) in parallel against *P. falciparum* PanK and the two CoA-pathway unrelated *P. falciparum* kinases, hexokinase (HK) and choline kinase (ChK). None of the investigated kinases were inhibited significantly at 2 μM, a concentration that is active in whole cell assays. At 10 μM, STK 668036 inhibited HK by 40 ± 2% (*t*-test *t* = 10.47, *df* = 10, *P* < 0.0001) and at 100 μM it showed significant 105 ± 1%, 84 ± 4% and 28 ± 8% inhibition on HK, PanK and ChK, respectively (ANOVA: *F*_(3,15)_ = 296.4, *P* < 0.001; Additional file 1: Figure S2b).

#### Rescue of CoA pathway metabolites in *P. falciparum* asexual stages

The asexual blood stage inhibitory activity of the compounds was compared with the activity obtained in the presence of the CoA biosynthesis pathway metabolites.

The most active compound on asexual *P. falciparum*, Amb180780, was rescued to approximately 100% of control levels by supplementation of dP-CoA or CoA and to 76 ± 8% by pantethine addition (ANOVA: *F*_(4,15)_ = 145.2,  *P* < 0.001), but not by pantothenate (Additional file 1: Table S2). This rescue pattern is consistent with the target of Amb180780 being either PPCS or PPCDC. Our pantothenate kinase assay (see above) did not reveal inhibition of PanK, thus suggesting that the compound is not a non-competitive inhibitor of PanK.

The growth inhibitory action of all the remaining compounds was rescued by supplementation of either dP-CoA or CoA, but not pantothenate or pantethine (Additional file 1: Table S2). This is consistent with the target of these compounds being towards the end of the CoA synthesis chain, namely inhibition of PPAT or competitive inhibition of DPCK. An alternative hypothesis could be competitive inhibition of some yet unidentified, downstream enzymes that utilize CoA. Supplementing additional CoA might overcome the competitive inhibition by the compound and thus restore function. The growth inhibitory effect of MMV000570 at 1 μM concentration could not effectively be rescued by any of the intermediates in this study. Since we were unable to reproduce the CoA rescue we previously observed for this compound [22], the compound’s activity is not discussed further, however all data for this compound can be found in the supplementary data section (Additional file 1: Table S3).

#### Rescue of CoA pathway metabolites in sexual *P. falciparum* stages

The ability of the metabolites to rescue the parasites from compound-mediated inhibition was then determined for early stage gametocytes for the compounds that had displayed sub-micromolar activity. The most active compound, Amb180780 (early gametocyte IC_50_ = 60 nM), was rescued to near 100% (ANOVA: *F*_(3,12)_ = 605.4, *P* < 0.0001; Tukey’s multiple comparisons test *P* < 0.001) of control levels by addition of pantethine, dP-CoA and CoA, supporting the observation with asexual stages that the compound is a PPCS or PPCDC inhibitor (Additional file 1: Table S2).

The rescue pattern for MMV665820 and Amb3377585 showed that their activity could only be counteracted by supplementation of dP-CoA and CoA (ANOVA: *F*_(2,9)_ = 65.66, *P* < 0.0001 and *F*_(2,9)_ = 75.44, *P* < 0.0001, respectively; Tukey’s multiple comparisons test *P* < 0.001 for both), confirming our finding in asexual blood stage *P. falciparum*. The most likely targets for these two compounds are therefore PPAT or competitive inhibition of DPCK (Additional file 1: Table S2).

STK 740987-treated early stage gametocytes (IC_50_ = 430 nM) showed low, albeit statistically significant levels of 23 ± 1% and 24 ± 1% rescue under dP-CoA and CoA supplementation (ANOVA: *F*_(2,13)_ = 441.5, *P* < 0.0001; Tukey’s multiple comparisons test *P* < 0.001), respectively, and were not rescued by the other intermediates (Additional file 1: Table S2). Since this compound showed high activity (> 95%) at its previously determined IC_80_ value, the rescue experiment was repeated whilst reducing the test concentrations to its IC_50_ value. This resulted in higher rescue levels of 46 ± 2% and 62 ± 1% under dP-CoA and CoA supplementation (ANOVA: *F*_(2,9)_ = 37.95, *P* < 0.001), respectively, whilst compound action remained unaffected by supplementation of pantothenate and pantethine. The observed rescue pattern indicates that this compound acts either on PPAT or as a competitive inhibitor of DPCK (Additional file 1: Table S2), although it might possess additional, gametocyte-specific target(s) outside the CoA synthesis pathway that can therefore not be rescued by CoA metabolites.

For the three most active compounds identified for late stage gametocytes, Amb4317088 (IC_50_ = 2.2 μM), STK740987 (IC_50_ = 460 nM) and Amb180780 (IC_50_ = 70 nM), the rescue pattern for the CoA pathway metabolites was also assessed. Amb4317088-treated late stage gametocytes were successfully rescued to above 100% of untreated control levels by supplementation of either CoA or dP-CoA (ANOVA: *F*_(2,11)_ = 1,932, *P* < 0.001; Tukey’s multiple comparisons test *P* < 0.001; Additional file 1: Table S2), thus confirming PPAT or DPCK as putative targets, as postulated regarding the other two parasite stages tested.

Similar to the observations in asexual blood stages and early stage gametocytes, STK740987 activity was counteracted in both dP-CoA and CoA-supplemented samples, albeit to lower rescue levels of approximately 50% (ANOVA: *F*_(2,11)_ = 289.8, *P* < 0.001; Tukey’s multiple comparisons test *P* < 0.001; Additional file 1: Table S2). This is consistent with the compound targeting either PPAT or DPCK and possibly the presence of an additional late stage gametocyte specific target.

Interestingly, Amb180780 treatment was rescued by dP-CoA and CoA (ANOVA: *F*_(2,11)_ = 823.7, *P* < 0.001; Tukey’s multiple comparisons test *P* < 0.001), but not by pantethine addition in late stage gametocytes, in contrast to asexual and early stage gametocyte cultures (Additional file 1: Table S2), possibly indicating a lack of uptake or utilization of pantethine in these late gametocyte development stages.

#### Rescue of CoA pathway intermediates in *T. b. brucei*

To ascertain whether the active compounds, Amb4317088, Amb3377585 and STK740987, affected the CoA synthesis pathway in *T. b. brucei*, supplementation of the pathway’s end-product, CoA, was tested. For all three compounds, CoA supplementation was able to rescue trypanosome numbers to between 70 ± 11% and 95 ± 4%, compared to untreated control values (ANOVA *F*_(2,13)_ = 284.9, 101.5 and 391.5, respectively, *P* < 0.001;  Additional file 1: Table S2). This observation confirms that all three inhibitors act on the CoA synthesis pathway or CoA utilization in *T. b. brucei*, as previously demonstrated for *P. falciparum* [22], and that this pathway offers opportunity for trypanocidal drug discovery.

As observed in *P. falciparum*, the growth-inhibitory activity of Amb4317088 and STK 740987 could also be rescued by supplementation of dP-CoA, but none of the other intermediates (Additional file 1: Table S2), indicating PPAT or competitive inhibition of DPCK as the most likely target candidates in *T. b. brucei*. For Amb3377585, besides the rescue by CoA and dP-CoA, pantothenate supplementation showed a slight, yet statistically significant (within 99.9% CI using Tukey’s multiple comparison test) 34 ± 1% rescue of *T. b. brucei* parasite numbers, whilst no pantothenate rescue had been observed for this compound in *P. falciparum*.

### Cytotoxicity

An initial indication of compound selectivity over human cells had previously been obtained through comparison of the compounds’ IC_50_ values against *P. falciparum* asexual stages and HEK293 [22]. In the present study, the selectivity investigation was extended to a small panel of human cancer cell lines, namely the estrogen receptor (ER)-positive breast cancer cell line MCF7, the ER-negative MDA-MB-231, as well as the human prostate cancer line, PC-3. Additionally, the cytotoxic effects on mouse embryonic fibroblast cell line 3T3, which serves as the host cell for *T. cruzi* in our image-based amastigote assay, were investigated [35].

Three of the compounds, namely MMV665980, STK 740987 and Amb180780 showed no or only negligible cytotoxicity against any of the cell lines investigated in this study, up to the highest tested concentration of 40 μM (Additional file 1: Table S4). These compounds had not shown cytotoxicity against HEK293 cells and, with the exception of the weak MMV665980, all the compounds had shown high activity against *P. falciparum* asexual blood stages and high selectivity indices in our previous study [22]. Of the remaining compounds, Amb3377585 and STK 668036 showed moderate inhibitory activity against all the cancer cell lines examined, with IC_50_ ranges of 5–9 μM and 3–5 μM (Additional file 1: Table S4). Although these compounds both inhibited HEK293 cells with similar potency in our previous study, they maintained acceptable parasite selectivity indices of 18 and 9, respectively [22].

The compounds Amb4317088 and MMV665820 displayed significant activity against the panel of cancer cell lines tested in this study, with IC_50_ values ranges of 0.87–4.6 μM and 0.27–0.43 μM, respectively (Additional file 1: Table S4). The inhibition plateau levels of the concentration-response curves for the latter compound, however, were well below 100% inhibition (ranging between 43% in MDA-MB-231 cells and 80% in PC-3 cells; Additional file 1: Figure S3). When only a sub-lethal plateau is reached, the calculated flex point of the concentration-response curve does not represent the concentration at which 50% of cells are inhibited, therefore it does not represent the true IC_50_ value of compound activity. This was previously observed in HEK293 cells, as well, and calculated IC_50_ values for this compound are to be considered approximates. Amb4317088 and MMV665820 had previously shown negligible or minor selectivity for *P. falciparum* over HEK293 cells, with SI values of 1.9 and ~6, respectively [22], thus are not considered worthwhile candidates to pursue.

The determination of cytotoxicity against the mouse 3T3 cells, necessary for the validation of the *T. cruzi* amastigote assay data, showed a similar sensitivity profile of these cells to that of the HEK293 reference cells published earlier [22], with the exception of STK 668036, to which the 3T3 mouse cells showed a lower susceptibility than any other cell line tested.

## Discussion

The open access MMV malaria box, a collection of diverse compounds with reported inhibitory activity against *P. falciparum* asexual stages, has been tested against several specific *Plasmodium* targets and pathways [40–43]. Furthermore, the collection was re-purposed to test efficacy of the compounds against a broad variety of parasites, including other apicomplexan parasites such as *Cryptosporidium parvum* and *Toxoplasma gondii* [44], the kinetoplastids *T. b. rhodesiense*, *T. b. brucei* and *T. cruzi* [45], the amoebozoa *Entamoeba histolytica* [37] and even helminths, such as *Schistosoma mansoni* [46]. This broad availability of species-specific data for a small set of compounds is unprecedented, and gives researchers the opportunity to identify activity patterns across multiple species and assays.

To directly assess the species-specificity of the previously-identified CoA pathway inhibitors (two from the MMV malaria box and five from a small in-house compound library [22]), we tested the same batch of compounds on *T. cruzi* and *T. b. brucei* using our previously reported assays*.* Trypanosomes are clinically important flagellate protozoan parasites of the order kinetoplastida, which are not closely related to the apicomplexan *P. falciparum*. In addition, we tested the compounds against a small panel of human cancer cell lines. In recent years, numerous reports have shown that compounds with antimalarial activity may represent potential anticancer agents, and vice versa [47–49]. The results obtained were compared to our previous findings on asexual *P. falciparum* and HEK293 cells [22]*.* The extended cytotoxicity pattern on the three cancer cell lines generally matched the previous observations on HEK293 cells. Our results suggest a lack of specific anticancer activity by the compounds (Additional file 1: Table S4).

For compound MMV665820 it was observed that the plateau for the CRC curves for all cell lines investigated were significantly below 100% (Additional file 1: Figure S3). This could indicate that the cytotoxic effect only impacts on a certain sub-population of these cells, perhaps as the result of a redundant process existing in mammalian cells, which allows a percentage of the inhibitor-treated cells to survive. The plateau for *P. falciparum* growth inhibition is observed at 100% [22], demonstrating that the asexual blood-stage parasite forms have no mechanism that ensures survival of a subpopulation under MMV665820 treatment.

We observed that the extracellular trypomastigote forms of both *T. b. brucei* and *T. cruzi* were more sensitive to compound treatment than the intracellular amastigote stage of *T. cruzi*. Further research will be needed to identify which of many possible reasons cause this different sensitivity. One possible explanation is that CoA synthesis could be more important in the trypomastigote life-cycle stage. Another possibility is that the additional barrier of the mammalian host cell surrounding the amastigote form might reduce compound penetration, and therefore result in a lower observed compound activity in the *T. cruzi* amastigote assay. Alternatively, the amastigote stage may be able to scavenge CoA from the host cell and thus be insensitive to the compound activity.

To investigate a potential role of CoA biosynthesis for host to vector transmission of the malaria parasite, we investigated the activity of our compounds on *P. falciparum* gametocytes, the sexual stages of *P. falciparum*, essential for malaria transmission. In addition to activity on the asexual blood stages, gametocytocidal activity is considered highly desirable for any new drug candidate to progress in the antimalarial drug development pipeline [50]. Parasite stage-specificity and gametocytocidal activity of the compounds was therefore investigated, utilizing a luciferase-based approach on early stage (development from stage I to IIb/III [30]) and late stage (IV to V [51]) gametocytes.

The same luciferase-based assays utilized in this study were previously used to assess the anti-gametocyte activity of the MMV malaria box compounds [30, 51]. In our prior work we used a screening concentration of 5 μM and a threshold of 50% activity to select hits to be progressed for dose-response evaluation. Of the two malaria box compounds tested in this work, MMV665980 had a gametocytocidal activity lower than the hit threshold, and was therefore not previously tested in dose-response. The other compound, MMV665820, had previously shown a similar weak inhibition against late stage gametocytes, and about 50% inhibition at 1 μM against early stage gametocytes in dose-response, thus in range (about 2.3-fold change) with the present study.

Whilst most of the compounds investigated showed similar activities against asexual blood stage *P. falciparum* and early stage gametocytes, two compounds, STK740987 and Amb180780, were additionally able to inhibit the viability of late stage gametocytes with similar sub-micromolar potency. Another compound, Amb4317088, showed low micromolar IC_50_ values against all three development stages tested. Our observations are consistent with active CoA synthesis being required in all three *P. falciparum* development forms investigated. The lower susceptibility of late stage gametocytes to most of the compounds, however, suggests that different levels of CoA synthesis or utilization are required during the progression of *P. falciparum* sexual development. This is consistent with metabolomic studies showing important metabolic differences between asexual and gametocyte stages [28], as well as during the course of gametocyte development [27]. Late stage gametocytes might have a lower basal requirement for CoA synthesis and/or utilization, either due to their terminal differentiation or potentially due to previous accumulation of sufficient CoA for maintenance of most of their metabolic functions. Alternatively, the late stage gametocyte or its RBC host cell might experience changes in membrane permeability affecting the uptake and/or efflux of some inhibitors. However, even late stage gametocytes were unable to tolerate 72 h exposure to effective CoA synthesis inhibitors like STK740987 and Amb180780, indicating that these compounds have a promising transmission blocking potential. Additionally, these two compounds displayed a favorable low cytotoxicity profile for all mammalian cell lines tested in our study. It would therefore be of interest to confirm the transmission blocking ability of these compounds by experimentally infecting mosquitoes with inhibitor-treated gametocytes (membrane feeding assay). Our investigation of the deformability of red blood cells infected with late stage gametocytes in conjunction with viability data, suggested that the observed deformability changes were either coincident with parasite death or a secondary effect of killing. Therefore, we could not find evidence of an independent effect on RBC deformability due to treatment with the CoA pathway inhibitors.

To probe directly the inhibitory activity of the compounds on the first step of the *P. falciparum* CoA synthesis pathway, we have utilized a radiometric phosphorylation assay, which allows measurement of pantothenate phosphorylation by PanK present in *P. falciparum* lysates [16, 39]. Only one compound, STK 668036, showed a statistically significant PanK inhibition of 93 ± 9% at a very high compound concentration (100 μM), compared to the whole cell activity of 1.3 μM IC_50_ on asexual blood stage *P. falciparum*. Since the parasites treated with the compound were rescued by addition of CoA or dephospho CoA, this weak inhibition of PanK points to other enzyme(s) of the pathway being the main target(s) for the compound, whereas the observed activity on PanK was possibly a non-specific off-target effect observed only at the higher concentrations of compound. This hypothesis is also supported by our observation that at high concentrations STK 668036 also inhibited other kinases in *P. falciparum* lysates.

To allow the generation of hypotheses regarding the inhibitors’ possible targets, given the current absence of enzymatic assays to directly assess inhibition of the subsequent steps of the CoA biosynthesis pathway, we utilized several pathway intermediates (panthotenate, panthetine, dephospho CoA and the final product CoA) as rescuing agents in our rescue assay.

To be able to utilize an intermediate, the parasite first has to be able to take it up from the surrounding medium. Eukaryotic cells readily take up and transform the CoA precursor 4′-phosphopantetheine [52] into CoA. In the case of *P. falciparum*, this task is made more difficult by the fact that the parasite is surrounded by the outer RBC membrane and the parasitophorous vacuole membrane. Our experiments suggest that all three *P. falciparum* developmental stages examined are able to take up and utilize CoA, as well as dP-CoA, as demonstrated by the ability of these intermediates to rescue parasite growth in the presence of the investigated CoA pathway inhibitors.

Since *T. b. brucei* is not closely related to *P. falciparum*, it was important to confirm whether the active compounds were also affecting the CoA synthesis pathway in this parasite. The growth inhibitory effects of all three compounds that had shown high activity against *T. b. brucei* could be rescued by addition of both CoA and dP-CoA. This rescue pattern was the same as observed in *P. falciparum*, which implies that the same targets in the CoA synthesis pathway may be affected. The fact that the inhibitors are not equally active on the different parasite species could indicate a different accumulation of inhibitor by different parasites, or structural differences in their target enzymes.

We also utilized the dimer pantethine in our rescue experiments based on the hypothesis that *P. falciparum* might possess a similar by-pass of its CoA synthesis pathway as bacteria, which are able to convert pantethine to pantetheine and further to 4′phosphopantetheine [38]. The fact that the growth inhibitory action of the test compound Amb180780 was successfully rescued by pantethine supplementation supports this hypothesis. Our findings are the first preliminary indication that asexual *P. falciparum* parasites, like bacteria, are able to take up and utilize the pantethine to synthesize CoA.

Early stage gametocytes treated with Amb180780 were also rescued by pantethine supplementation, but late stage gametocytes were not. It seems unlikely that the target of the compound would change between the two gametocyte development stages, therefore this lack of responsiveness to pantethine could indicate that the late stage gametocytes are either unable to take up pantethine from the medium or that they lack the ability to convert it into the intermediate 4′phosphopantetheine. None of the compounds that showed activity against *T. b. brucei* were rescued by pantethine supplementation. However, the activity of the same compounds was also not susceptible to pantethine supplementation in *P. falciparum.* Therefore, no conclusion can be drawn at this stage regarding whether trypanosomes are able to utilize pantethine for CoA synthesis.

We also observed that notably higher pantethine concentrations were tolerated by early and late stage gametocytes (up to 10 mM concentration showing no toxic effect) compared to asexual blood stage *P. falciparum* (IC_50_ value of 5 mM) or *T. b. brucei* (IC_50_ value of 1.2 mM). It would be interesting to investigate further the mechanism of pantethine uptake in the different *P. falciparum* development stages, since a potential variation in the uptake or elimination mechanism for this intermediate could also affect the transmembrane transport of other molecules. Reduced sensitivity of gametocytes to compounds active against blood-stage *P. falciparum* parasites has frequently been observed [29, 30, 53], therefore the investigation of stage specific differences, as observed here, might reveal novel routes for targeted intervention.

Interestingly, a recent study on dihydroartemisinin-induced dormant ring stages of *P. falciparum* showed that these metabolically inactive parasite forms still maintain active fatty acid synthesis [54]. This observation implies that such dormant ring forms are likely to have a continuing requirement for CoA, which is an essential cofactor in fatty acid synthesis. This opens the possibility that potent, specific *P. falciparum* CoA synthesis inhibitors could in future be utilized to help to reduce the recrudescence from dormant parasite forms. Such an application of *P. falciparum* CoA synthesis inhibitors could have clinical implications, since dormant parasite forms are currently an important concern regarding the failure of artemisinin combination therapies [54].

Pantothenate uptake is essential for asexual *P. falciparum* survival [55]. In our investigation, none of the tested compounds were rescued by pantothenate addition, excluding the possibility of the compounds being competitive inhibitors of pantothenate transport, while non-competitive inhibition remains a possibility.

The evidence provided in this work suggests that the investigated compounds affect *P. falciparum* and *T. b. brucei* CoA biosynthesis, albeit with various degrees of specificity, warranting further confirmation. The chemical diversity of the compounds is surprising if a single target pathway is hypothesized. Recent evidence, however, has shown that antimalarial compounds belonging to chemically unrelated classes may share the same molecular target [56–59].

Recombinant expression of the CoA synthesis enzymes would be of great value for use in specific enzyme-coupled activity assays to test compounds directly. Having the molecular structures of these enzymes would also allow the use of computational modeling and docking studies, to gain more detailed insight into the compound-target interactions and to facilitate inter-species comparison.

## Conclusions

In this work we carried out a biological profiling of previously identified asexual *P. falciparum* stage inhibitors against the sexual stages of the parasite, as well as the kinetoplastid parasites *T. cruzi* and *T. b. brucei* and cancer cell lines. We demonstrate for the first time that the malaria parasite’s gametocytogenesis, like its asexual replication, relies on CoA. Two of the candidate compounds exhibited sub-micromolar potencies across all *Plasmodium* stages tested. Furthermore, three compounds inhibited the viability of *T. cruzi* and *T. b. brucei* trypomastigotes with sub-micromolar potencies. By exposing the parasites to various intermediates of the CoA synthesis pathway, we could confirm that the active compounds act on *P. falciparum* and *T. b. brucei* CoA synthesis, and provided support for the hypothesis that the CoA synthesis pathway is a valuable new drug target in these parasite species.

## Methods

### Chemical compounds

The compounds MMV665820 and MMV665980 are part of the Medicines for Malaria Venture (MMV) Malaria Box, kindly provided by MMV. The compounds Amb4317088 (Ambinter), STK 668036 (Interchim), STK 740987 (Vitas M), Amb3377585 (Ambinter) and Amb180780 (Ambinter) were originally identified from the screening of a library consisting of structurally diverse, commercially available compounds. For this work, solids were repurchased from various suppliers, as indicated in brackets above. The chemical structures of the study compounds are shown in Additional file 1: Table S1. All compounds were prepared as 10 mM 100% DMSO stocks, from which the compounds were diluted to the respective final assay concentration (see below for each assay).

### Cytotoxicity assay

Cytotoxicity of the compounds was assessed against a panel of human cancer cell lines, namely the estrogen receptor (ER)-positive breast cancer cell line MCF7, the ER-negative MDA-MB-231 and the prostate cancer line PC-3. All the cell lines were purchased from ATCC and maintained as previously described [60, 61]. Two biological replicates consisting of two technical replicates each were performed to generate concentration response curves (CRC) with a final highest concentration of 40 μM. A resazurin-based assay was utilized to determine cell viability as described earlier [22]. Additionally, the cytotoxic effects on the mouse embryonic fibroblast cell line 3T3 were assessed as part of the *Trypanosoma cruzi* amastigote image-based assay as outlined in [35].

### *Trypanosoma cruzi* amastigote assay

Compound activity against *T. cruzi* amastigotes was assessed in an image-based assay in single point concentration-response and three biological replicates, at a final highest compound concentration of 47.3 μM, as previously described [35].

### *T. cruzi* trypomastigote assay

Compound activity against *T. cruzi* trypomastigotes was assessed in two independent experiments, without technical repeats, at a final highest compound concentration of 40 μM in concentration-response PrestoBlue assay, as previously described [35].

### *Trypanosoma brucei brucei* assay

Compound activity against *T. b. brucei* was assessed in a resazurin-based viability assay in concentration-response assays, without technical repeats, in two experimental replicates at a highest concentration of 40 μM, as previously described [36].

### Gametocyte luciferase assay

Induction of *P. falciparum* gametocyte differentiation and gametocyte culturing were performed as previously described [62]. Compound activity against early stage gametocytes (covering the development from stage I to IIb/III) and late stage gametocytes (stage IV to V) of the recombinant *P. falciparum* line NF54^Pfs16^ was assessed in concentration-response assays, in two experimental replicates consisting of two technical repeats each, at a highest concentration of 40 μM, as described earlier [30, 51].

### Deformability of gametocyte-infected red blood cells

Compound impact on the deformability profile of red blood cells (RBCs) infected with stage V gametocytes of the recombinant *P. falciparum* line NF54^Pfs16^ was assessed in two biological replicates in two technical repeats each, using an optimized method of that previously described by Duez et al. [34]. In short, calibrated microspheres of sizes 25–45 μm (type 6) and 5–15 μm (type 3) (solder powder AMTech®,

Deep River, CT, USA) were each mixed separately in PBS (Sigma-Aldrich, Sydney, Australia) supplemented with 10 mg/ml Albumax II (Life Technologies, Carlsbad, CA, USA) and were sequentially deposited into 384 well filter bottom plates (Seahorse Bioscience, North Billerica, MA, USA). Stage V gametocyte cultures at day 11 post-induction (24 h incubation) or day 12 post-induction (2 h incubation), obtained as previously described [62], were dispensed into 384 well plates (Axygen®) at 0.5% hematocrit and ~5% gametocytemia and incubated at 37 °C with 90% N_2_, 5% CO_2_, 5% O_2_ with compounds for the indicated exposure durations. Compounds were tested in concentration-response assays at a final highest concentration of 40 μM, in two biological replicates, without technical repeats. Post incubation, samples were resuspended and loaded into microsphere filter plates and vacuum aspirated into the microsphere matrix. Then, wash buffer (gametocyte growth medium without N-acetyl-D-glucosamine) was added followed by vacuum aspiration into a 384 well storage plate. After microsphere filtration, *c*.100,000 cells/well were transferred to imaging plates (Perkin Elmer cell carrier) containing PBS and CellMask™ orange 1:15,000 (Invitrogen, Carlsbad, CA, USA) and imaged 12–24 h later using an Opera confocal high content imaging system (Perkin Elmer) with 20× magnification. Ten fields per well were acquired resulting in > 10,000 RBCs/well being analysed using Opera® Columbus software (to quantify RBCs and gametocytes). Retention rates were calculated as described previously [34].

### Pantothenate kinase assay

The ability of the test compounds to inhibit *P. falciparum* pantothenate kinase (*Pf*PanK) was assessed with a radiometric pantothenate phosphorylation assay as described previously [39]. In short, the phosphorylation of [^14^C] pantothenate by *P. falciparum* lysates was measured in the presence or absence of a single concentration (100 μM, two biological replicates in duplicate technical repeats) of each test compound. Additionally, the activity of STK 668036 on *Pf*PanK and two additional parasite kinases, namely hexokinase (*Pf*HK), and choline kinase (*Pf*ChK), was tested at 2, 10 and 100 μM compound concentration, in two independent experiments. *P. falciparum* lysate was prepared from trophozoite stage parasites isolated from their host RBC by treatment with 0.05% (w/v) saponin, and washed (three times) in HEPES-buffered saline (125 mM NaCl, 5 mM KCl, 20 mM D-glucose, 25 mM HEPES, 1 mM MgCl_2_, pH 7.1). Lysates were prepared in 10 mM potassium phosphate, pH 7.4, the concentration of cell lysates was determined from cell counts with an improved Neubauer hemocytometer, and aliquots of lysate were stored at -20 °C.[^14^C] pantothenate, [^14^C] 2-deoxyglucose and [^14^C] choline phosphorylation were assayed using Somogyi reagent as described earlier [39].

### Chemical rescue with CoA pathway intermediates

The *P. falciparum* luciferase and *T. b. brucei* assays described above were modified by adding CoA pathway metabolites (pantothenate, pantethine, dP-CoA and CoA) to allow assessment of chemical rescue. An imaging based assay was utilised to assess the chemical rescue on asexual stages of *P. falciparum* as described previously [22]. All the pathway intermediates were purchased from Sigma. CoA and dP-CoA were dissolved in sterile distilled water to obtain a 20 mM stock solution, which was then aliquoted and stored at -20 °C until use. Pantothenate and pantethine were prepared freshly before use from solids. Each pathway intermediate was tested on the parasites at a range of concentrations to assess potential toxicity. The final concentration ranges were 10 mM to 78 μM in 8 serial dilution steps for pantothenate; 10 mM to 1.2 μM in 16 serial dilution steps for pantethine; 2 mM to 0.6 mM in 8 dilutions steps of 0.2 mM increments for CoA. On early stage *P. falciparum* gametocytes the same concentrations as for CoA were tested for dP-CoA; against asexual blood stage *P. falciparum*, late stage gametocytes and *Trypanosoma b. brucei* five dP-CoA concentrations were tested (2 mM, 1.5 mM, 1 mM, 0.75 mM and 0.5 mM).

The same concentration ranges of the CoA pathway intermediates were supplemented to parasites treated with the experimental compounds. The inhibitors were used at their respective final assay concentration of IC_80_ (as determined previously in the corresponding *P. falciparum* development stage or *T. b. brucei* assay). Due to varying toxicity and effectiveness of the intermediates on the various parasites and life-cycle stages tested, the best rescue concentration was determined individually for each intermediate on each parasite or parasite stage. The concentrations that provided the best rescue results (as reported in Additional file 1: Table S2) were the following: Pantothenate, 300 μM on asexual *P. falciparum* blood stages, 625 μM on early and 312.5 μM on late stage gametocytes, 1.25 mM on *T. b. brucei*; pantethine, 125 μM on asexual *P. falciparum* blood stages, 10 mM on early and 5 mM on late stage gametocytes, 2.44 μM on *T. b. brucei*; dP-CoA, 0.8 mM on asexual *P. falciparum* blood stages, 1.2 mM on early and 2 mM on late stage gametocytes, 1.6 mM on *T. b. brucei*; CoA 0.8 mM on asexual *P. falciparum* blood stages, 1.6 mM on early and 1.8 mM on late stage gametocytes, 1.0 mM on *T. b. brucei*. The negative control used in each assay was 0.4% DMSO, whilst the positive control was 2 μM artemisinin for asexual *P. falciparum* blood stages and 5 μM puromycin for all gametocyte stages and *T. b. brucei*. The percentage growth of the respective parasites following compound treatment was determined using Microsoft ® Excel 2010, and compared with their controls. All rescue assays were performed as two independent experiments each in duplicate point.

### Statistical analysis

Statistical analysis and graphical output were performed in GraphPad Prism® v.5. CRC fitting was carried out by employing a variable slope, 4 parameter non-linear regression analysis. One way ANOVA and Tukey’s multiple comparison test were used to assess the significance of the rescue with supplementation of metabolites versus compound treatment alone.

## Additional file

Additional file 1:  Figure S1. Retention of gametocyte-infected RBCs after treatment for 2 h (light grey dots) or 24 h (dark grey triangles) with compound concentrations ranging from 10 μM to 0.1 nM. Viability of compound treated gametocytes assessed with Mitotracker Red (MTR), shown as black squares. For all tested compounds, any increased retention rates observed coincided with parasite death, therefore no specific RBC deformability change due to compound treatment could be determined. **Figure S2.** Effect of test compounds on kinase activity using a radiometric assay (*n* = 2). (A) Activity of all compounds on PanK at 100 μM; (B) activity of STK668036 on three kinases: PanK, Hexokinase (HK) and Choline kinase (ChK). Asterisks indicate a significant level of inhibition, compared to the control (*P* < 0.001). Error bars represent standard deviations. **Table S1.** Activity overview against *P. falciparum* asexual blood stage forms, early and late stage gametocytes, as well as *T. b. brucei* and *T. cruzi*. **Table S2.** Rescue of inhibitor-treated asexual blood stage *P. falciparum*, early stage and late stage *P. falciparum* gametocytes and *T. b. brucei* trypomastigotes by supplementation with CoA pathway intermediates. Percent rescue averages ± SEM. The rescue experiment was not carried out for inactive compounds (blank spaces). All numeric values are statistically significant (*P* < 0.001). **Table S3.** Activity of MMV000570 against *Trypanosoma* spp., cytotoxicity against a panel of cell lines and selectivity indices (A); the activity of MMV000570 against *P. falciparum* asexual stages and early stage gametocytes could not be rescued by CoA pathway metabolites; percent rescue averages ± SEM (B). **Table S4.** Cytotoxicity of test compounds on human cancer cell lines in comparison with reference cell lines and antiplasmodial activity. **Figure S3.** Concentration response curves displaying cytotoxic effect of MMV665820 on the four mammalian cell lines MCF7 (A), MDA-MB-231 (B), PC3 (C) and 3 T3 (D). (PDF 905 kb)

## Abbreviations

ACT: Artemisinin combination therapies
ATP: Adenosine triphosphate
CoA: Coenzyme A
CRC: Concentration response curves
DMSO: Dimethylsulphoxide
DPCK: Dephospho CoA kinase
dP-CoA: Dephospho coenzyme A
ER: Estrogen receptor
IC_50_: Half maximal inhibitory concentration
MMV: Medicines for Malaria Venture
PanK: Pantothenate kinase
*Pf*ChK: *P. falciparum* choline kinase
*Pf*HK: *P. falciparum* hexokinase
*Pf*PanK: *P. falciparum* pantothenate kinase
PPAT: Phosphopantetheine adenylyltransferase
PPCDC: Phosphopantothenoylcysteine decarboxylase
PPCS: Phosphopantothenoylcysteine synthetase
RBC: Red blood cell
SI: Selectivity index
TCA: Tricarboxylic acid
