# Supplementary material for: Biological characterization of chemically diverse compounds targeting the Plasmodium falciparum coenzyme A synthesis pathway
Source: Parasit Vectors. 2016 Nov 17;9:589. doi: 10.1186/s13071-016-1860-3 (PMC5114727; doi:10.1186/s13071-016-1860-3)
Supplement: Additional file 1: Figure S1. — Retention of gametocyte-infected RBCs after treatment for 2 h (light grey dots) or 24 h (dark grey triangles) with compound concentrations ranging from 10 μM to 0.1 nM. Viability of compound treated gametocytes assessed with Mitotracker Red (MTR), shown as black squares. For all tested compounds, any increased retention rates observed coincided with parasite death, therefore no specific RBC deformability change due to compound treatment could be determined. Figure S2. Effect of test compounds on kinase activity using a radiometric assay (n = 2). (A) Activity of all compounds on PanK at 100 μM; (B) activity of STK668036 on three kinases: PanK, Hexokinase (HK) and Choline kinase (ChK). Asterisks indicate a significant level of inhibition, compared to the control (P < 0.001). Error bars represent standard deviations. Table S1. Activity overview against P. falciparum asexual blood stage forms, early and late stage gametocytes, as well as T. b. brucei and T. cruzi. Table S2. Rescue of inhibitor-treated asexual blood stage P. falciparum, early stage and late stage P. falciparum gametocytes and T. b. brucei trypomastigotes by supplementation with CoA pathway intermediates. Percent rescue averages ± SEM. The rescue experiment was not carried out for inactive compounds (blank spaces). All numeric values are statistically significant (P < 0.001). Table S3. Activity of MMV000570 against Trypanosoma spp., cytotoxicity against a panel of cell lines and selectivity indices (A); the activity of MMV000570 against P. falciparum asexual stages and early stage gametocytes could not be rescued by CoA pathway metabolites; percent rescue averages ± SEM (B). Table S4. Cytotoxicity of test compounds on human cancer cell lines in comparison with reference cell lines and antiplasmodial activity. Figure S3. Concentration response curves displaying cytotoxic effect of MMV665820 on the four mammalian cell lines MCF7 (A), MDA-MB-231 (B), PC3 (C) and 3 T3 (D). (PDF 905 kb) [file 13071_2016_1860_MOESM1_ESM.pdf]

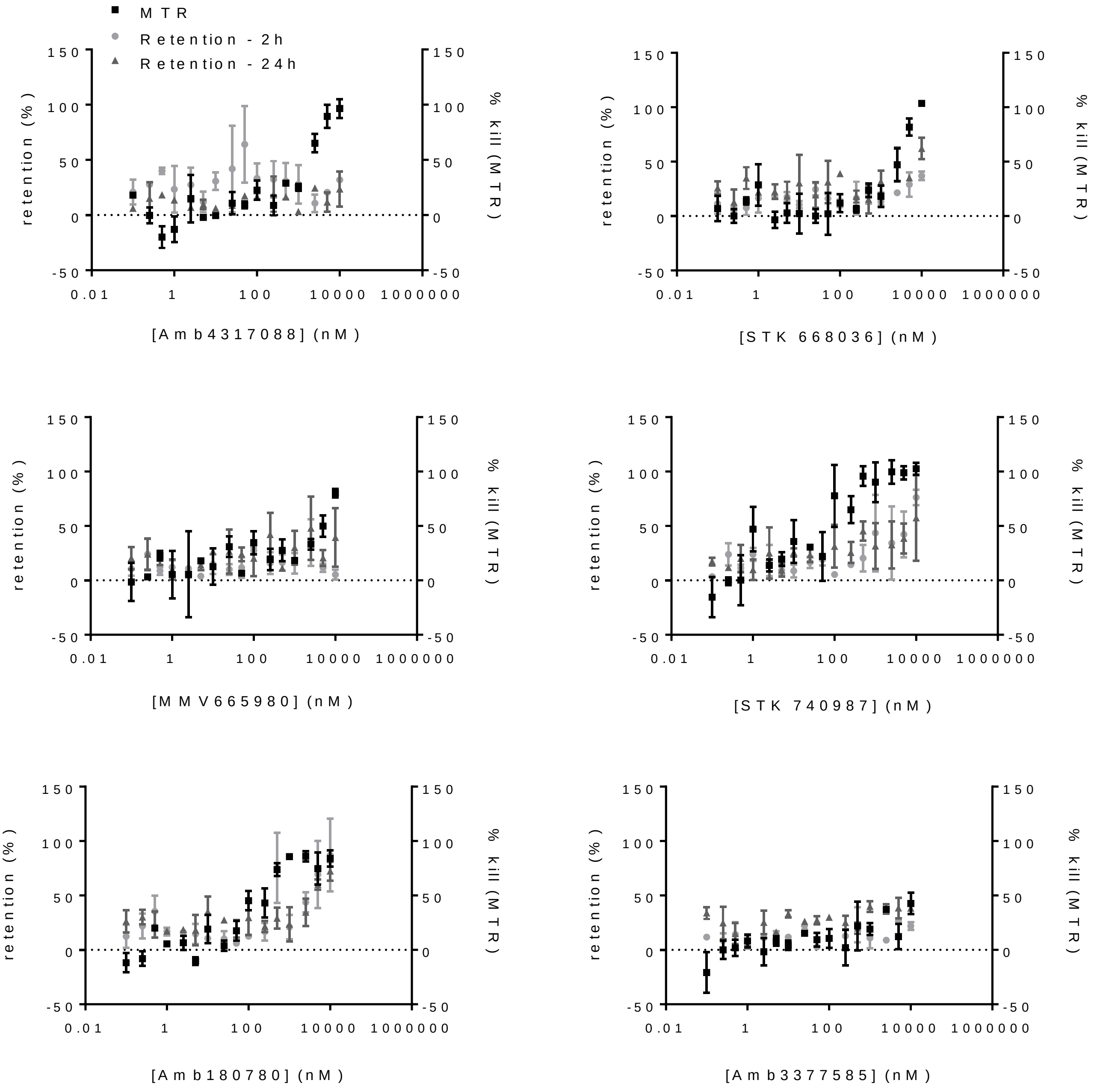

Figure S1: Retention of gametocyte infected RBCs after treatment for 2 h (light grey dots) or 24 h (dark grey triangles) with compound concentrations ranging from 10 µM to 0.1 nM. Viability of compound treated gametocytes assessed with Mitotracker Red (MTR), shown as black squares. For all tested compounds, any increased retention rates observed coincided with parasite death, therefore no specific RBC deformability change due to compound treatment could be determined.

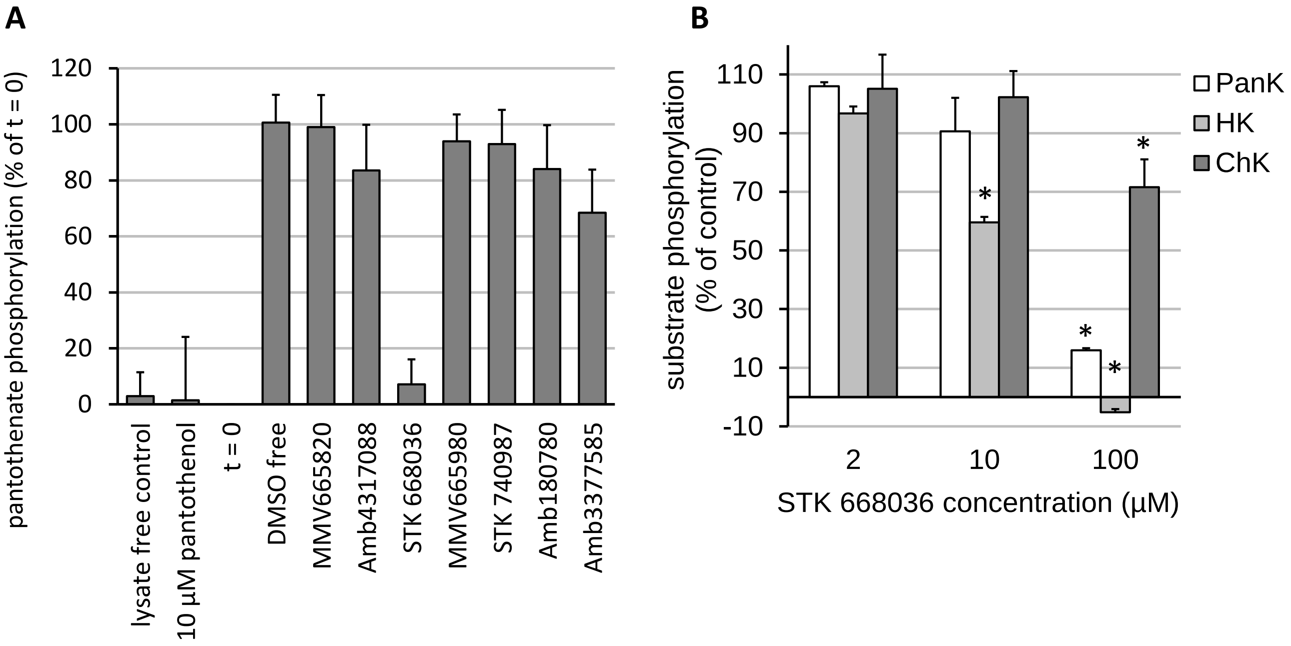

Figure S2: Effect of test compounds on kinase activity using a radiometric assay (n = 2).
(A) Activity of all compounds on PanK at 100 µM; (B) activity of STK668036 on three kinases: PanK, Hexokinase (HK) and Choline kinase (ChK). Asterisks indicate a significant level of inhibition, compared to the control ($p < 0.001$). Error bars represent standard deviations.

Table S1: Activity overview against *P. falciparum* asexual blood stage forms, early and late stage gametocytes, as well as *T. b. brucei* and *T. cruzi*.

		**Activity on *Trypanosoma* spp. IC_{50} ± SEM (µM)[a]**			**Activity on *P. falciparum* life stages IC_{50} ± SEM (µM) [a]**		
**Chemical structure**	**compound ID**	***T. b. brucei* trypomastigote (SI over HEK293)**	***T. cruzi* amastigote (SI over 3T3 host cell)**	***T. cruzi* trypomastigote (SI over HEK293)**	**Asexual[b]**	**Early stage gametocyte**	**Late stage gametocyte**
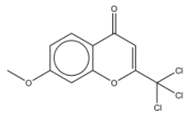	MMV665820 / 5534045	2.50 ± 1.66 (nd[d])	62%[c] (nd[d])	100% (nd[d])	0.58 ± 0.08	0.44 ± 0.03	69%
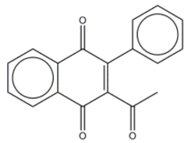	Amb4317088	0.16 ± 0.01 (15)	4.30 ± 0.27 (2)	0.39 ± 0.03 (6)	1.23 ± 0.06	2.88 ± 0.18	2.24 ± 0.38
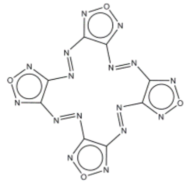	STK 668036	100% (nd[d])	94% (nd[d])	2.07 ± 0.46 (6)	1.30 ± 0.08	1.56 ± 0.12	3.53 ± 0.38
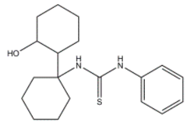	MMV665980/ Amb2368822	100% (nd[d])	70% (nd[d])	4.42 ± 0.89 (> 9)	6.31 ± 1.06	7.54 ± 0.39	4.15 ± 0.56
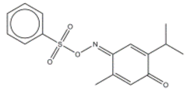	STK 740987	1.02 ± 0.40 (> 39)	54% (nd[d])	0.68 ± 0.15 (> 59)	0.38 ± 0.06	0.43 ± 0.06	0.46 ± 0.12

Chemical structure	compound ID	Activity on *Trypanosoma* spp. IC_{50} ± SEM (μM)[a] *T. b. brucei* trypomastigote (SI over HEK293)	*T. cruzi* amastigote (SI over 3T3 host cell)	*T. cruzi* trypomastigote (SI over HEK293)	Activity on *P. falciparum* life stages IC_{50} ± SEM (μM)[a] Asexual[b]	Early stage gametocyte	Late stage gametocyte
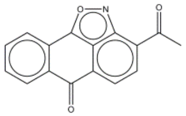	Amb3377585	0.20 ± 0.01 (14)	13.30 ± 2.35 (1)	0.74 ± 0.13 (4)	0.15 ± 0.01	0.25 ± 0.01	6.22 ± 2.5
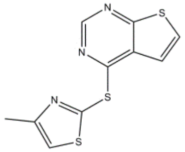	Amb180780	100% (nd[d])	21% (nd[d])	100% (nd[d])	0.12 ± 0.02	0.06 ± 0.004	0.07 ± 0.01

[a]Percent inhibition at the highest concentration tested (40 μM) is given if an IC_{50} value could not be calculated due to low activity of the compound

[b]As previously reported (1)

[c]Low plateau level of CRC curve in cytotoxicity assay

[d]An accurate IC_{50} value could not be calculated

Abbreviations: nd, not determined; SI, The selectivity indices of compound activity against *T. b. brucei* and *T. cruzi* trypomastigote forms was calculated over HEK293 cytotoxicity [22] and of *T. cruzi* amastigote forms over 3T3 host cell cytotoxicity

1. **Fletcher S, Avery VM.** 2014. A novel approach for the discovery of chemically diverse anti-malarial compounds targeting the Plasmodium falciparum Coenzyme A synthesis pathway. Malar J **13:**343.

Table S2: Rescue of inhibitor-treated asexual blood stage *P. falciparum*, early stage and late stage *P. falciparum* gametocytes and *T. b. brucei* trypomastigotes by supplementation with CoA pathway intermediates. Percent rescue averages ± SEM. The rescue experiment was not carried out for inactive compounds (blank spaces). All numeric values are statistically significant ($P < 0.001$)

**Enzyme**	**PanK**				**PPCS / PPCDC**				**PPAT / DPCK**				**Complete pathway**			
Growth inhibition rescued by	Panthothenate (competitive inhibitors)				Pantethine				Dephospho CoA				CoA			
stage / species	PfAS	PfEG	PfLG	Tbb	PfAS	PfEG	PfLG	Tbb	PfAS	PfEG	PfLG	Tbb	PfAS	PfEG	PfLG	Tbb
Amb180780	No	No	No		76.2 ± 7.7	94.2 ± 1.8	No		99.3 ± 3.8	104.6 ± 0.5	95.2 ± 1.3		113.5 ± 4.4	111.3 ± 3.2	105.7 ± 2.9	
MMV665820/ 5534045	No	No			No	No			88.6 ± 18.3	98.7 ± 1.9			113.3 ± 12.3	98.8 ± 3.0		
Amb4317088	No		No	No	No		No	No	77.6 ± 5.5		107.9 ± 1.7	84.7 ± 2.6	84.5 ± 7.7		128.5 ± 2.6	85.0 ± 6.9
STK 668036	No				No				81.7 ± 5.4				82.9 ± 4.8			
Amb2368822/ (MMV665980)	No				No				46.0 ± 2.6				54.4 ± 2.5			
STK 740987	No	No	No	No	No	No	No	No	88.9 ± 6.7	23.2 ± 1.2 (46.2 ± 2.4)[a]	50.3 ± 0.8	90.3 ± 5.3	103.9 ± 3.4	24.1 ±0.7 (61.5 ± 1.0)[a]	53.1 ± 0.5	69.6 ± 10.6
Amb3377585	No	No		34.2 ± 1.3	No	No		No	38.7 ± 4.7	89.5 ± 0.3		100.4 ± 1.4	89.3 ± 2.6	103.4 ± 1.8		95.0 ± 4.2

Abbreviations: PfAS, *Plasmodium falciparum* asexual blood stages; PfEG, *P. falciparum* early stage gametocytes (stage I-III); PfLG, *P. falciparum* late stage gametocytes (stages IV-V); Tbb, *Trypanosoma brucei brucei*

[a]percent rescue at the compound's IC_{50} is shown in brackets

Table S3: Activity of MMV000570 against *Trypanosoma* spp., cytotoxicity against a panel of cell lines and selectivity indices (A); the activity of MMV000570 against *P. falciparum* asexual stages and early stage gametocytes could not be rescued by CoA pathway metabolites; percent rescue averages ± SEM (B).

A

**Activity on *Trypanosoma ssp.*; IC_{50} [µM] ± SEM†**			**Cell line specific cytotoxicity; IC_{50} [µM] ± SEM†**				
***T. b. brucei* trypomastigote (SI over HEK293)**	***T. cruzi* amastigote (SI over 3T3 host cell)**	***T. cruzi* trypomastigote (SI over HEK293)**	**HEK293***	**3T3**	**MCF7**	**MDA-MB-231**	**PC-3**
5.85 ± 1.11 (~ 6.8)	96% (ND)	100% (ND)	~ 40	> 40	96%	90%	98%

† Percent inhibition at the highest concentration tested (40 µM) is given if an IC_{50} value could not be calculated due to low activity of the compound

SI: The selectivity indices of compound activity against *T. b. brucei* and *T. cruzi* trypomastigote forms was calculated over HEK293 cytotoxicity and of T. cruzi amastigote forms over 3T3 host cell cytotoxicity.

* As previously reported (1)

B

**enzyme**	**PanK**					**PPCS / PPCDC**					**PPAT / DPCK**					**Complete pathway**				
Growth inhibition rescued by	Panthothenate					Pantethine					Dephospho CoA					CoA				
stage‡ (replicate #)	PfAS (1)	PfAS (2)	PfAS (3)	PfEG (1)	PfEG (2)	PfAS (1)	PfAS (2)	PfAS (3)	PfEG (1)	PfEG (2)	PfAS (1)	PfAS (2)	PfAS (3)	PfEG (1)	PfEG (2)	PfAS (1)	PfAS (2)	PfAS (3)	PfEG (1)	PfEG (2)
tested at IC_{50} concentration	3.5 ± 1.3	-0.6 ± 0.8		4.3 ± 0.4	28.6 ± 3.8	3.5 ± 0.5	1.4 ± 1.5		5.1 ± 0.2	20.9 ± 3.2	2.3 ± 0.4	2.7 ± 1.4		15.6 ± 0.5	59.0 ± 2.4	3.8 ± 0.8	4.7 ± 3.4		17.5 ± 1.0	61.3 ± 2.1
tested at IC_{80} concentration	3.1 ± 1.4	2.2 ± 1.3	2.3 ± 2.3	5.2 ± 1.1		2.1 ± 0.8	1.2 ± 0.8	0.1 ± 1.6	5.5 ± 1.2		4.6 ± 0.9	6.3 ± 2.4	1.2 ± 1.3	26.0 ± 1.3		6.4 ± 1.2	8.1 ± 2.1	-1.6 ± 1.3	26.7 ± 1.5	

‡ PfAS: *Plasmodium falciparum* asexual blood stages; PfEG: *P. falciparum* early gametocytes (stage I-III)

1. **Fletcher S, Avery VM.** 2014. A novel approach for the discovery of chemically diverse anti-malarial compounds targeting the Plasmodium falciparum Coenzyme A synthesis pathway. Malar J **13:**343.

Table S4: Cytotoxicity of test compounds on human cancer cell lines in comparison with reference cell lines and antiplasmodial activity.

Compound ID	Asexual *P. falciparum*; IC_{50} [µM] ± SEM*	Cell line specific cytotoxicity; IC_{50} [µM] ± SEM†				
		HEK293* (SI)	3T3	MCF7	MDA-MB-231	PC-3
MMV665820 / 5534045	0.58 ± 0.08	3.6[a] ± 0.24 (ND)	0.47[a]	0.27[a] ± 0.05	0.43[a]	0.35[a] ± 0.01
Amb4317088	1.23 ± 0.06	2.33 ± 0.12 (1.9)	8.6 ± 0.96	4.6 ± 0.16	0.87 ± 0.04	1.4 ± 0.06
STK 668036	1.30 ± 0.08	11.92 ± 1.32 (9.2)	> 20	5.5 ± 0.21	> 4	3 ± 0.12
MMV665980/ Amb2368822	6.31 ± 1.06	> 40 (> 6)	> 40	> 40	> 40	> 20
STK 740987	0.38 ± 0.06	> 40 (> 105)	> 20	> 40	> 40	> 20
Amb3377585	0.15 ± 0.01	2.75 ± 0.18 (18.3)	13.4[a] ± 1.95	9.1 ± 0.62	5.6 ± 0.33	5.5 ± 0.64
Amb180780	0.12 ± 0.02	~ 40 (~ 333)	> 40	> 40	> 40	> 40

IC_{50}: inhibitory concentration 50%
SEM: standard error of mean
SI: selectivity index.
† the highest concentration resulting in < 50 % inhibition is given if the inhibition plateau was not reached by 40 µM and an IC_{50} value could therefore not be accurately calculated; it is inferred that the actual IC_{50} must be higher than the given values of < 50% inhibition.
* As previously reported (1).
[a] Low plateau level of concentration-response curve does not allow determination of accurate IC_{50}.
ND: could not be determined.

1. **Fletcher S, Avery VM.** 2014. A novel approach for the discovery of chemically diverse anti-malarial compounds targeting the Plasmodium falciparum Coenzyme A synthesis pathway. Malar J **13:**343.

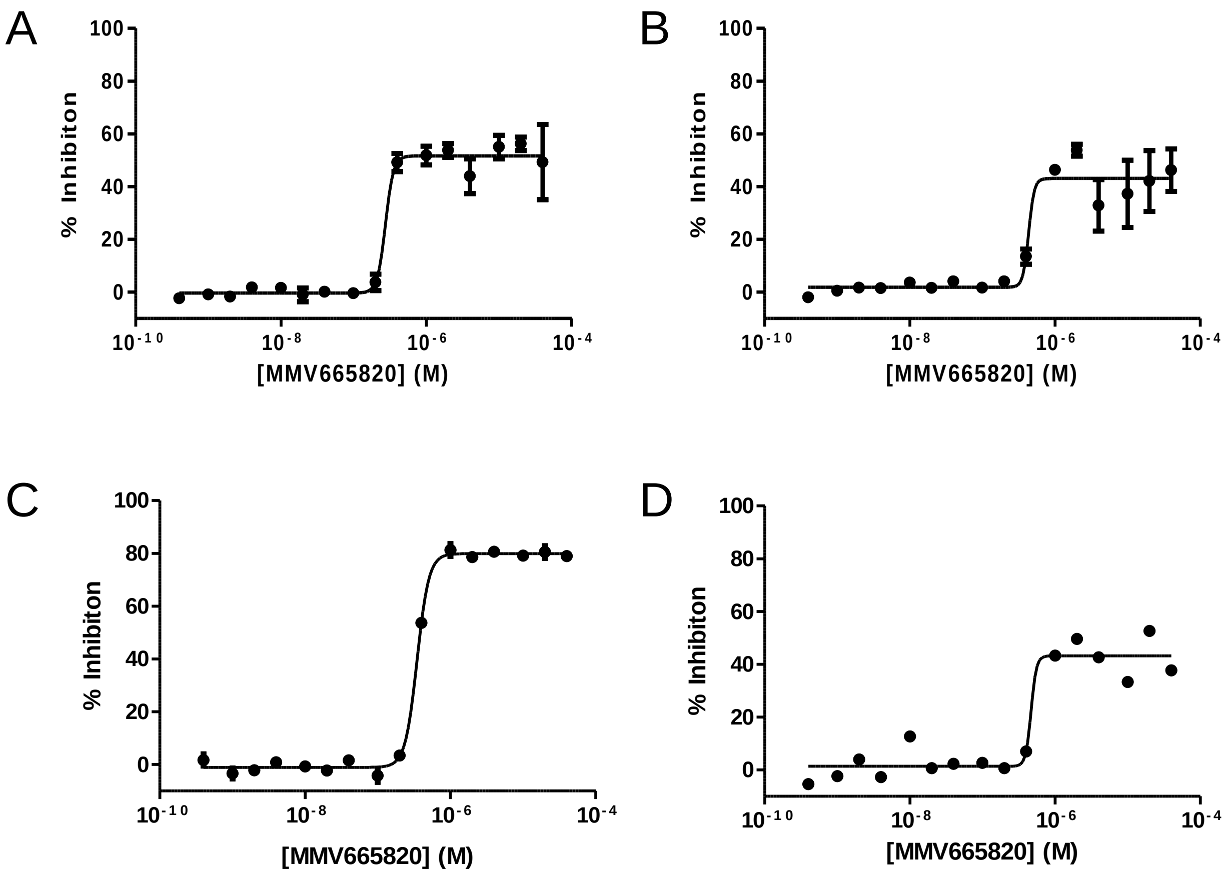

Figure S3: Concentration response curves displaying cytotoxic effect of MMV665820 on the four mammalian cell lines MCF7 (A), MDA-MB-231 (B), PC3 (C) and 3T3 (D).
